# Laser in situ keratomileusis versus Artisan lens implantation in correcting ametropia after penetrating keratoplasty for keratoconus

**DOI:** 10.1186/s12886-023-02848-x

**Published:** 2023-03-17

**Authors:** Sepehr Feizi, Mohammad Ali Javadi, Niloufar Bineshfar, Hamed Esfandiari

**Affiliations:** Ocular Tissue Engineering Research Center, Research Institute for Ophthalmology and Vision Science, Shahid Beheshti University of Medical Sciences, No. 23, Paidarfard St., Boostan 9 St., Pasdaran Ave, Tehran, Iran

**Keywords:** Laser in situ keratomileusis, Artisan phakic intraocular lens, Penetrating keratoplasty, Keratoconus, Refractive error

## Abstract

**Purpose:**

To compare the long-term safety and efficacy of laser in situ keratomileusis (LASIK) with Artisan phakic intraocular lens implantation to correct refractive errors after penetrating keratoplasty (PK) for keratoconus.

**Methods:**

This retrospective comparative interventional case series included a total of 33 consecutive keratoconus eyes that had previous PK and received subsequent LASIK (*n* = 16) or Artisan lens implantation (*n* = 17) were included in this study. Outcome measures were uncorrected distance visual acuity (UDVA), corrected distance visual acuity (CDVA), refractive error, and complications.

**Results:**

Postoperatively, the UDVA of ≥20/40 was achieved in none of the LASIK group compared to 62.5% of eyes in the Artisan group (*P* < 0.001); the respective values for CDVA of ≥20/40 were 87.5 and 94.1% (*P* = 0.51). Spherical equivalent refraction decreased from − 6.97 ± 1.50 D preoperatively to − 4.20 ± 2.05 D postoperatively in the LASIK group (*P* < 0.001) and from − 10.79 ± 2.15 D preoperatively to − 2.13 ± 1.23 D postoperatively in the Artisan group (*P* < 0.001). There was no significant change in the refractive astigmatism in LASIK group (*P* = 0.30) or Artisan group (*P* = 0.11). The efficacy and safety indices were significantly better for Artisan (0.82 ± 0.34 and 1.13 ± 0.30, respectively) than for LASIK (0.22 ± 0.17 and 0.85 ± 0.24, respectively, *P* ≤ 0.006 for both comparisons). While refractive error changed significantly from postoperative year 3 to the final visit in the LASIK group, it remained stable in the Artisan group through follow-up period. No significant complications were observed in any group.

**Conclusion:**

Artisan lens implantation provided superior and stable visual outcomes compared to LASIK for the management of post PK refractive errors in keratoconus eyes.

## Key summary points

### Why carry out this study?

- Refractive error is the most common complication after penetrating keratoplasty performed in keratoconus eyes.
- A variety of surgical alternatives such as laser in situ keratomileusis and phakic intraocular lens implantation are available for correcting post keratoplasty refractive error.
- However, there is no study that compared clinical outcomes after these procedures in keratoconus patients who received corneal transplantation.

### What was learned from the study?

- Artisan phakic intraocular lens implantation yields better and stable long-term visual and refractive outcomes compared to laser in situ keratomileusis for the treatment of ametropia after penetrating keratoplasty for keratoconus.
- Artisan intraocular lens implantation should be the primary technique for correction of refractive error for this category of patients when contact lenses or glasses correction is not tolerated.

## Introduction

The safety and efficacy of penetrating keratoplasty (PK) in advanced keratoconus is well established, and its outcomes have been enhanced further by the improvement in the surgical technique and tissue banking [1]. However, clear PK graft does not necessarily lead to better visual function, which could be compromised by postsurgical refractive errors [1]. Spectacles and contact lenses are the first step in the management of ametropia after corneal transplantation [2]. However, post-keratoplasty refractive error cannot always be corrected by spectacles due to high degrees of astigmatism, myopia, and anisometropia, whereas contact lens intolerance may prevent its use due to lack of motivation, poor manual dexterity, blepharitis, dry eye, and steep corneal graft. A variety of surgical alternatives are available for correcting post keratoplasty refractive error. Arcuate keratotomy, relaxing incisions with compression sutures, and wedge resection have been shown to reduce post-graft refractive errors. These procedures are unpredictable and mainly reduce the astigmatic component of the refractive error, with no significant effect on the spherical component [2, 3].

Other surgical methods, including photorefractive keratectomy (PRK), laser in situ keratomileusis (LASIK), and phakic intraocular lens (IOL) implantation, have been used for the management of post-PK ametropia. PRK’s outcomes are less predictable, it may not completely correct the refractive error, and has a relatively high rate of complications such as subepithelial haze formation, regression, and irregular astigmatism [2, 4, 5]. LASIK procedure treats a wider range of refractive error after corneal transplantation and offers several advantages over PRK including faster visual recovery, minimal regression, and reduced stromal opacification [6–20]. However, its use is limited by the graft stromal thickness and amount of refractive error suitable for correction. In addition, complications such as flap buttonhole, wound dehiscence due to the high vacuum pressure, and epithelial ingrowth may occur with LASIK. Phakic IOL implantation is another surgical option for the management of post PK refractive errors. A few studies have demonstrated the safety and efficacy of phakic IOL to correct spherocylindrical refractive error after PK [21–24]. However, there is no study that compared clinical outcomes after LASIK versus phakic IOL implantation in keratoconic patients who received PK. Herein, we aim to compare the long-term efficacy, accuracy, safety, and stability of LASIK versus Artisan spherical iris-fixated phakic IOL implantation in correcting myopic astigmatism in eyes with prior PK for keratoconus.

## Methods

The protocol of this study was approved by the Institutional Review Board at the Shahid Beheshti University of Medical Sciences and adhered to the tenets of the Declaration of Helsinki guidelines for human research.

Thirty-three consecutive eyes of 33 patients with previous PK who underwent LASIK or Artisan IOL implantation between April 15, 2008 and May 10, 2019 were included in this retrospective comparative case series. All the enrolled eyes had received PK for the management of severe keratoconus and were not satisfied with spectacles correction due to high compound myopic astigmatism and were contact lens intolerant. Inclusion criteria consisted of age ≥ 21 years at the time of refractive surgery, clear corneal graft that had been transplanted at least 18 months before the refractive surgery, absence of other ocular pathologies (dry eye, uveitis, cataract, glaucoma, or retinal pathologies), a preoperative corrected distance visual acuity (CDVA) of ≥20/50, regular and symmetric astigmatism by tomography (Orbscan II system, Bausch & Lomb, Rochester, New York, USA), and documented stable refractive error, defined as a change ≤0.50 D in sphere, ≤1.00 D in astigmatism magnitude, or ≤ 10° in astigmatism orientation for at least 6 months after complete suture removal or placement of relaxing incision for astigmatism correction. None of the eyes had wound override, posterior donor-recipient junction gape, or uneven wound healing. Eyes with less than 1 year of follow-up after refractive surgery were excluded.

Sixteen eyes underwent LASIK and 17 received Artisan spherical phakic IOL implantation (Artisan, Ophtec BV, Groningen, the Netherlands). The surgical method was determined based on the amount of ametropia, thinnest corneal pachymetry, and anticipated residual stromal bed thickness. LASIK was offered for preoperative spherical equivalent between − 4 and − 10 D, refractive astigmatism ≤ − 6 D, a thinnest corneal thickness > 500 μm, and anticipated residual stromal bed thickness > 300 μm and > 50% of the preoperative pachymetric reading after the full correction of refractive error. Artisan phakic IOL implantation was preferred when LASIK was contraindicated due to inadequate preoperative corneal thickness for full refractive error correction and a high amount of spherical equivalent (> − 10 D). Preoperative requirements for Artisan implantation included anterior chamber depth ≥ 3.0 mm (measured from the endothelium to the crystalline lens), endothelial cell count ≥1500 cells/mm^2^, refractive astigmatism ≤ − 4 D, and mesopic pupil diameter < 6 mm for myopia ≤ − 15.50 D and < 5 mm for myopia > − 15.50 D. We performed relaxing incision located at the donor-recipient junction with concomitant counterquadrant compression sutures to reduce preoperative refractive astigmatism to ≤6 D in the LASIK group and to ≤4 D in the Artisan group. This procedure was repeated if the sufficient correction of astigmatism was not achieved after the first operation.

### Preoperative examination

Preoperative examination included uncorrected distance visual acuity (UDVA), CDVA, manifest refraction, cycloplegic refraction, keratometry readings, slit-lamp biomicroscopy, applanation tonometry, and indirect ophthalmoscopy. An autokeratorefractometer (software version 1.2.6; Carl Zeiss Meditec AG) was used to objectively measure manifest refraction. Subjective refraction was determined by a single experienced optometrist using the highest plus technique. Orbscan II system was used to measure corneal graft curvature and thickness and anterior chamber depth in all eyes. In Artisan group, graft endothelial cell evaluation was done preoperatively using a noncontact specular microscope (Topcon SP-3000P; Topcon Corporation, Tokyo, Japan).

### Surgical techniques

All the procedures were performed by a single experienced surgeon (M.A.J.). The target refraction in all patients was set at emmetropia, and the attempted correction was chosen on the basis of manifest refraction. LASIK procedure was performed under topical anesthesia using a standardized one-stage technique. A suction ring was centered on the pupil at the limbus and a microkeratome (Hansatome; Bausch & Lomb Surgical, Claremont, CA) was used to create a 160-μm-thick lamellar flap. The lamellar cut had a diameter of 9.0 mm, more than the size of the graft, with a superior hinge. Keracor Technolas 217 C (Bausch & Lomb Surgical, Claremont, CA) was used to ablate the stromal bed, with an ablation zone diameter of 5 mm. The flap was then replaced in its original position, the interface was copiously irrigated using balanced salt solution to prevent flap folds and interface debris, and its border was carefully dried with microsponges. No enhancement procedure was performed in LASIK-treated eyes.

The Artisan implantation procedure was done under general anesthesia. The dioptric power of the lens was determined on the basis of manifest spherical equivalent refraction, anterior chamber depth, and keratometry readings. The diameter of the lens optic was 5 mm for myopia > − 15.50 D and 6 mm for myopia ≤ − 15.50 D. A two-plane, posterior limbal incision was created at 12-o’clock, followed with two paracenteses at 2- and 10-o’clock. Acetylcholine and cohesive viscoelastic material were injected into the anterior chamber, then the Artisan lens was inserted into the anterior chamber and rotated to the horizontal position. The center of the lens was adjusted over the center of the pupil, and a bite of the iris stroma was enclavated into lens claws, using an enclavation needle. A peripheral iridotomy was performed at 12-o’clock to prevent pupillary block. The viscoelastic material was thoroughly irrigated and replaced with balanced salt solution, and the incision was sutured using 10–0 nylon sutures. The tension of the sutures was adjusted with standard qualitative Maloney keratoscope.

### Postoperative course

Postoperative examinations were performed at 1 day, 1 week, 1, 3, and 6 months, and every 6 months thereafter. We performed UDVA measurement, biomicroscopic examination, and intraocular pressure measurement at all examinations. Manifest refraction, CDVA, and keratometry readings were recorded after postoperative month-1. Postoperative treatment in both groups included topical chloramphenicol 0.5% and topical betamethasone 0.1% every 6 hours for 1 week. After a week, chloramphenicol 0.5% was discontinued, whereas betamethasone 0.1% was slowly tapered off over 2 to 3 months. Beginning at week 4, separate sutures were selectively removed from the Artisan-implanted eyes over a period of 2 months, depending on the subjective refraction; all sutures were removed by postoperative month 3. Specular microscopy was performed at 12-month postoperative visit in the Artisan group.

### Outcome measures and statistics

Postoperative visual and refractive outcomes were compared between the two groups after complete suture removal in Artisan eyes. Efficacy of each procedure was defined as reduction in refractive error and safety was defined based on losing ≥2 lines of CDVA. The efficacy index was defined as the mean postoperative UDVA divided by the mean preoperative CDVA. The safety index was defined as the mean postoperative CDVA divided by the mean preoperative CDVA. Other outcome measures included refractive stability over the course of the study and the rate of complications. The astigmatic results were analyzed both arithmetically (subtraction method) and with regard to the astigmatism axis using vector analysis. For vector analysis, polar (astigmatism magnitude and axis) values were converted to a Cartesian (x and y) coordinates. The coordinates were plotted in a double-angled plot, and the result was then reconverted into polar coordinates. The centroid was determined using the mean ± standard deviation value of the refractive cylinder.

Statistical analysis was performed using the SPSS, version 25 (SPSS Inc., Chicago, Illinois, USA). Snellen UDVA and CDVA were transformed to logarithm of the minimum angle of resolution (logMAR) values. The normality of the distribution of data was investigated using the Kolmogorov-Smirnov test. The paired t test was used to compare preoperative and postoperative data in each study group, and the two groups were compared in baseline and postoperative visual and refractive outcomes using two-sample t tests. We used Mann Whitney test to compare the mean number of Snellen lines gained/lost from preoperative to the last visit. A chi-square test was used to compare the two groups in distributions of UDVA, CDVA, and the number of lines gained/lost. To compare the refractive outcomes at different follow ups (baseline, 3, 6 months, and 1, 3 years, and last follow up), repeated-measures analysis of variance (ANOVA) with Bonferroni adjustment for the repeated measures was used. A *P* value < 0.05 was considered statistically significant.

## Results

The demographic and clinical characteristics including sex, age at the time of refractive surgery, the laterality of the operated eye, time interval from corneal transplantation and complete suture removal to refractive surgery, and follow-up duration were comparable between both groups (Table 1). Graft size was 8.11 ± 0.16 mm (range, 7.75 to 8.25 mm) and 8.15 ± 0.16 mm (range, 7.75 to 8.25 mm) in the LASIK and phakic IOL groups, respectively (*P* = 0.48). Five eyes (31.3%) in the LASIK group and 10 eyes (58.8%) in the phakic IOL group received relaxing incision and counterquadrant compression suture for high graft astigmatism after PK and prior to their refractive surgery (*P* = 0.11). Refractive surgery was deferred for 12.6 ± 7.3 months (9 to 21 months) and 9.1 ± 3.7 months (6 to 13 months) after relaxing incision in the LASIK and Artisan groups, respectively (*P* = 0.28).

**Table 1:** Comparison of demographic and baseline clinical characteristics between patients who received laser in situ keratomileusis versus Artisan lens implantation for the management of refractive error after penetrating keratoplasty for keratoconus

Parameters	LASIK group	Artisan group	*P* value
Sex (Male/Female)	9/7	11/6	0.62
Age (years)	30.8 ± 8.4 (21 to 51)	31.5 ± 5.4 (23 to 43)	0.79
Eye (Right/Left)	5/11	9/8	0.21
Graft size (mm)	8.11 ± 0.16 (7.75 to 8.25)	8.15 ± 0.16 (7.75 to 8.25)	0.48
Interval from penetrating keratoplasty to refractive surgery (months)	39.6 ± 23.1 (20 to 108)	56.2 ± 39.2 (18 to 122)	0.15
Interval from complete suture removal to refractive surgery (months)	24.0 ± 21.1 (6 to 93)	39.5 ± 36.6 (6 to 110)	0.14
Follow up (months)	75.6 ± 29.4 (29 to 135)	58.8 ± 35.3 (12 to 118)	0.15

*LASIK* Laser in situ keratomileusis

Patients in LASIK group had significantly better baseline UDVA, CDVA, and spherical equivalent refraction, while preoperative refractive and keratometric astigmatism was lower in Artisan group (Table 2). The study groups were comparable in preoperative mean keratometry (Table 2). None of the study eyes had preoperative UDVA ≥20/40. Preoperative CDVA ≥20/30 was observed in 81.3% of eyes in LASIK and 41.2% of eyes in Artisan group (*P* = 0.02).

**Table 2:** Comparison of preoperative and postoperative visual acuity, refraction, and keratometry readings between eyes that underwent laser in situ keratomileusis versus Artisan lens implantation for the management of refractive error after penetrating keratoplasty for keratoconus

Parameters		LASIK group	Artisan group	*P* value (between group comparison)
UDVA (LogMAR)	Preoperative	1.20 ± 0.32 (0.60 to 1.60)	1.48 ± 0.16 (1.30 to 1.70)	0.04
	Postoperative	0.92 ± 0.47 (0.40 to 1.60)	0.34 ± 0.17 (0.18 to 0.60)	0.007
	*P* value	0.42	0.001	
CDVA (LogMAR)	Preoperative	0.15 ± 0.10 (0.0 to 0.40)	0.24 ± 0.08 (0.08 to 0.30)	0.009
	Postoperative	0.22 ± 0.10 (0.10 to 0.40)	0.21 ± 0.08 (0.10 to 0.40)	0.72
	*P* value	0.04	0.24	
Spherical equivalent (D)	Preoperative	−6.97 ± 1.50 (−9.25 to −4.0)	−10.79 ± 2.15 (−14.0 to − 6.75)	< 0.001
	Postoperative	−4.20 ± 2.05 (− 9.25 to −2.0)	−2.13 ± 1.23 (− 4.25 to −0.38)	0.001
	*P* value	< 0.001	< 0.001	
Refractive astigmatism (D)	Preoperative	−3.41 ± 1.74 (−6.0 to 0.0)	−2.71 ± 1.51 (− 4.0 to 0.0)	0.03
	Postoperative	−4.28 ± 2.07 (−9.0 to −1.0)	−2.28 ± 1.50 (− 4.0 to 0.0)	0.003
	*P* value	0.30	0.11	
Mean keratometry (D)	Preoperative	46.91 ± 1.35 (45.0 to 49.0)	47.77 ± 2.94 (42.5 to 48.0)	0.34
	Postoperative	44.70 ± 2.62 (39.50 to 47.96)	46.55 ± 1.47 (44.0 to 47.75)	0.17
	*P* value	0.002	0.15	
Keratometric astigmatism (D)	Preoperative	2.94 ± 1.21 (1.50 to 5.07)	1.62 ± 1.84 (0.0 to 5.81)	0.04
	Postoperative	5.34 ± 2.18 (2.50 to 8.48)	2.70 ± 2.97 (1.0 to 6.50)	0.03
	*P* value	0.01	0.66	

*LASIK* Laser in situ keratomileusis, *UDVA* Uncorrected distance visual acuity, *CDVA* Corrected distance visual acuity, *LogMAR* Logarithm of minimum angle of resolution, *D* Diopter

### Visual acuity outcome

Postoperative visual acuity and refractive data were available for all eyes up to 12 months, 15 LASIK and 16 Artisan eyes at 18 months, 15 LASIK and 13 Artisan eyes at 2 years, and 14 LASIK and 12 Artisan eyes at 3 years. Follow-up of more than 3 years was available for 14 LASIK and 11 Artisan eyes. No significant difference was observed between baseline and postoperative UDVA in the LASIK group (Table 2). UDVA significantly improved in the Artisan group at the final follow up visit compared to baseline values (Table 2). Intergroup comparison revealed a significantly better postoperative UDVA in the Artisan group (Table 2). Postoperatively, the UDVA of ≥20/40 was achieved in none of the LASIK group compared to 62.5% of eyes in the Artisan group (*P* < 0.001). Fifty percent of the eyes in LASIK and 100% of the eyes in Artisan group had a postoperative UDVA ≥20/80, respectively (*P* = 0.01).

**Fig. 1:**
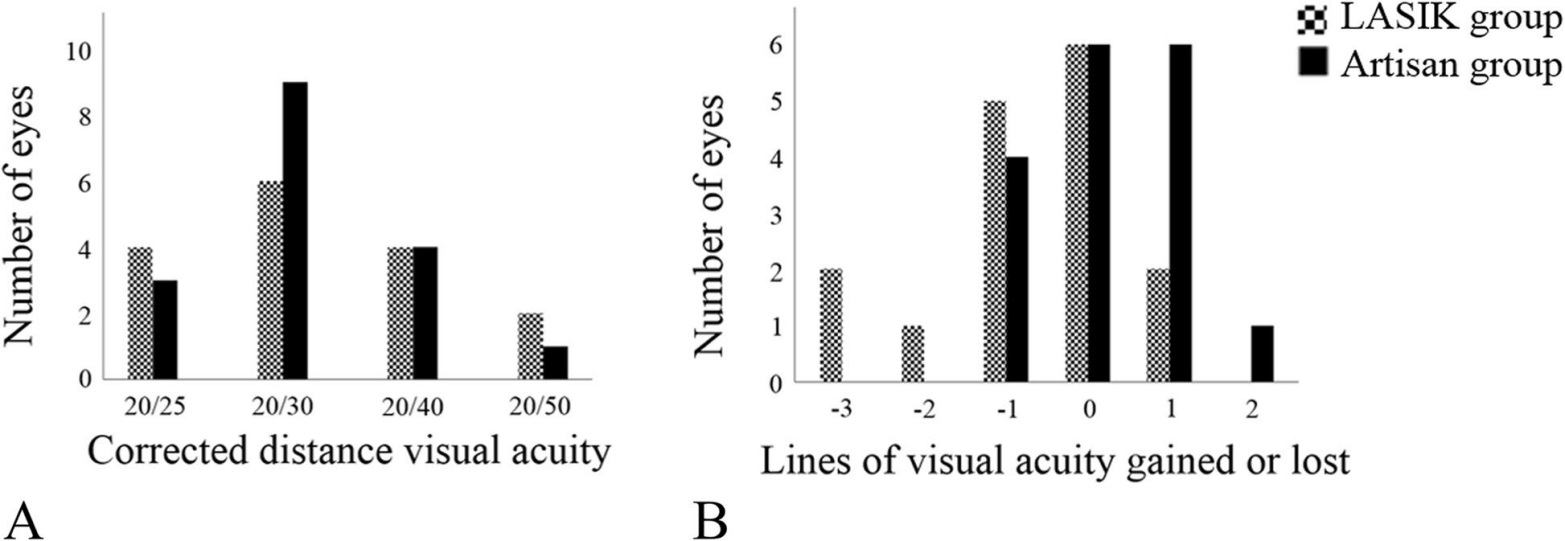
The bar graph demonstrates the distribution of corrected distance visual acuity (**A**) and number of gained or lost lines of corrected distance visual acuity (**B**) after laser in situ keratomileusis (LASIK) and Artisan lens implantation for the management of refractive error after penetrating keratoplasty for keratoconus

Compared to preoperative values, CDVA significantly decreased in the LASIK group at the last examination (Table 2). No significant difference, however, was observed between preoperative and postoperative CDVA in the Artisan group (Table 2). At the final follow-up visit, 87.5% in LASIK and 94.1% in Artisan group had postoperative CDVA ≥20/40 (*P* = 0.51, Fig. 1). The mean change in the lines of CDVA was − 0.94 ± 1.69 (range, − 4 to 2) and 0.56 ± 1.46 (range, − 2 to 3) in the LASIK and Artisan groups, respectively (*P* < 0.001). At the final follow-up, 37.5% of the eyes in LASIK group and 35.3% of the eyes in Artisan group maintained their preoperative CDVA (*P* = 0.90). Loss of ≥2 lines of CDVA was observed in 3 LASIK-treated eyes (18.8%) and no Artisan-implanted eye (*P* = 0.23). A gain of at least 1 line of CDVA was observed in 2 eyes (12.5%) in the LASIK group and 7 eyes (41.2%) in the Artisan group (*P* = 0.02, Fig. 1). The efficacy index was significantly better for Artisan than for LASIK (0.82 ± 0.34 versus 0.22 ± 0.17, respectively, *P* = 0.001). The safety index was significantly better for Artisan than for LASIK (1.13 ± 0.30 and 0.85 ± 0.24, respectively, *P* = 0.006).

### Refractive outcomes

**Fig. 2:**
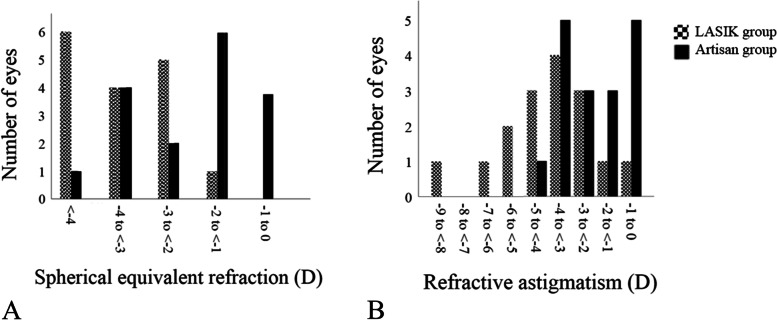
Distribution of spherical equivalent refraction (**A**) and refractive astigmatism (**B**) after laser in situ keratomileusis (LASIK) and Artisan lens implantation for the management of refractive error after penetrating keratoplasty for keratoconus

Spherical equivalent refraction was significantly decreased in both groups at the final follow-up visit (Table 2). Spherical equivalent decreased by 40.3% ± 22.2% in the LASIK group and 80.0% ± 11.2% in the Artisan group (*P* < 0.001). Postoperative spherical equivalent refraction ≤ − 1 D, − 2 D, − 3 D, and − 4 D was observed in 0% versus 23.5% (*P* = 0.17), 6.3% versus 58.8% (*P* = 0.04), 37.5% versus 70.6% (*P* = 0.06), and 62.5% versus 94.1% (*P* = 0.03) of the LASIK and Artisan groups, respectively (Fig. 2).

**Fig. 3:**
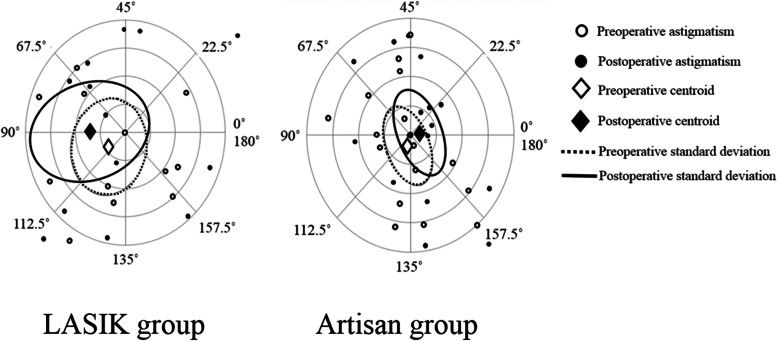
Double-angled plots of preoperative and postoperative astigmatism in eyes that underwent laser in situ keratomileusis (LASIK) and Artisan lens implantation for the management of refractive error after penetrating keratoplasty for keratoconus. Each concentric circle represents 1 D astigmatism magnitude. The ellipses surrounding the centroids indicate the standard deviations of the x and y coordinates

While the postoperative astigmatism was significantly lower in the Artisan group, refractive astigmatism did not change postoperatively in each group compared to the preoperative values. Postoperative refractive astigmatism ≤ − 1 D, − 2 D, − 3 D, and − 4 D was observed in 6.3% versus 29.4% (*P* = 0.09), 12.5% versus 47.1% (*P* = 0.03), 31.3% versus 64.7% (*P* = 0.05), and 56.3% versus 94.1% (*P* = 0.01) of the LASIK and Artisan groups, respectively (Fig. 2). The double-angled plots revealed that the centroid changed from 0.83 D @ 108° ± 3.24 D before surgery to 1.35 D @ 89° ± 4.22 D after LASIK and from 0.36 D @ 126° ± 2.45 D to 0.42 D @ 6° ± 2.60 D after Artisan implantation (Fig. 3). Analysis of the centroid suggests an increase in the magnitude of the astigmatism and standard deviation post LASIK but not after Artisan implantation. There was a shift in the axis of astigmatism in both groups. At the final follow-up visit, 14 patients (87.5%) in the LASIK group and 8 patients (47.1%) in the Artisan group needed eye glasses for residual refractive error (*P* = 0.01).

### Keratometry outcomes

Mean keratometry decreased while keratometric astigmatism increased significantly after LASIK (Table 2). These values remained unchanged in the Artisan group. There was no significant difference in mean keratometry values between the two groups, whereas keratometric astigmatism was significantly lower in the Artisan group at the final follow-up visit (Table 2).

### Refractive predictability and stability

**Fig. 4:**
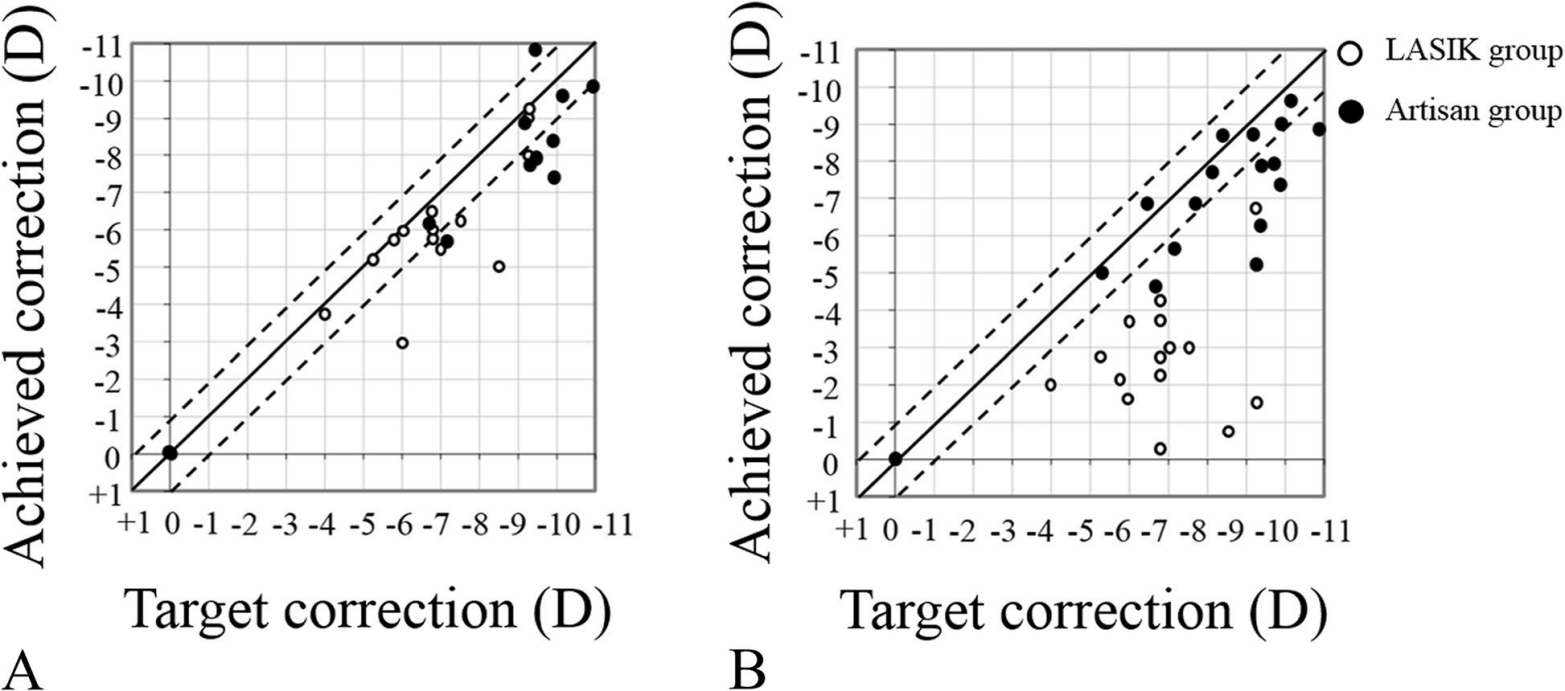
Scattergram of the target versus achieved correction of spherical equivalent refraction at postoperative month-3 (**A**) and final visit (**B**) in eyes that received laser in situ keratomileusis (LASIK) or Artisan lens implantation for the management of refractive error after penetrating keratoplasty for keratoconus. Dotted lines indicate ±1.0 diopter of emmetropia

**Fig. 5:**
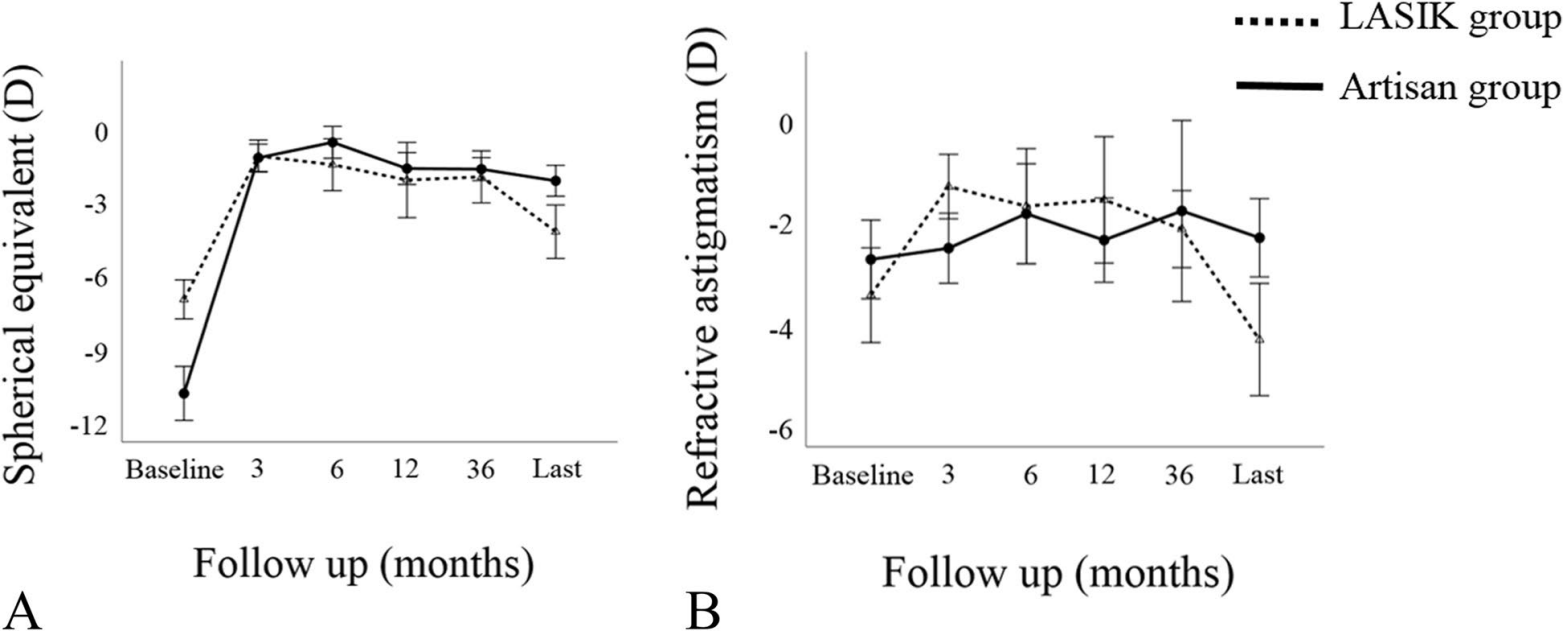
Changes in spherical equivalent refraction (**A**) and refractive astigmatism (**B**) through the course of the study after laser in situ keratomileusis (LASIK) and Artisan lens implantation for the management of refractive error after penetrating keratoplasty for keratoconus

The predictability of each procedure was investigated by evaluating the target versus achieved correction of the spherical equivalent refractive error at postoperative month 3 and final follow-up (Fig. 4). Considering stability, repeated-measures ANOVA disclosed a significant decrease in the spherical equivalent refraction and refractive astigmatism up to post-LASIK month-3 follow-up visit (*P* < 0.001 and *P* = 0.009, respectively). The values remained stable until year-3 follow-up visit (*P* > 0.20 for all comparisons, Fig. 5), then increased from year-3 to the final follow-up visit (*P* = 0.007 and 0.006, respectively). The mean change in spherical equivalent refraction and refractive astigmatism from postoperative month-3 to the final visit was 3.24 ± 1.69 D (range, 1.38 to 6.5 D) and 2.60 ± 2.01 D (range, − 0.75 to 6.75 D), respectively.

For Artisan group, there was a significant decrease in spherical equivalent refraction from baseline to 3 months postoperatively (*P* < 0.001). Afterwards, it remained unchanged until the final follow-up visit (*P* > 0.30 for all comparisons, Fig. 5). Refractive astigmatism did not change postoperatively compared to the baseline values (*P* = 0.61, Fig. 5). The mean change in spherical equivalent refraction and refractive astigmatism from postoperative month-3 to the final visit was 1.05 ± 1.47 D (range, − 1.63 to 3.63 D) and − 0.11 ± 0.96 D (range, − 2.0 to 1.25 D), respectively.

### Complications

No intra- or postoperative complication was observed in the LASIK group. On postoperative day-1, all eyes had a well-centered flap with intact epithelium. No adverse event of buttonhole flaps, wound dehiscence, diffuse lamellar keratitis, or epithelial ingrowth were encountered. In Artisan group, the phakic IOL was well-centered with round and reactive pupil. There was one case of uveitis after IOL implantation that was successfully treated with frequent topical corticosteroids. No case of high intraocular pressure or graft rejection was observed, and all the grafts maintained their optical clarity at the last examination. In the Artisan-implanted eyes, the endothelial cell count at the baseline and one-year follow-up was 1736 ± 301 cells/mm^2^ and 1602 ± 692 cells/mm^2^, respectively (*P* = 0.71).

## Discussion

This study compared the long-term outcomes of the LASIK and phakic IOL implantation in the management of post-PK refractive errors. Several studies have reported the outcomes of LASIK and phakic IOL implantation after keratoplasty [6–24]. However, none of these studies compared the outcomes of these two procedures. In our practice, Artisan IOL is offered to post-PK patients when LASIK is not indicated such as high levels of myopia and inadequate corneal graft thickness. This explains the statistically significant difference in preoperative UDVA and spherical equivalent refraction between the groups. We included patients who had chiefly regular symmetric astigmatism after corneal transplantation, based on preoperative CDVA and corneal tomography. The lower CDVA in Artisan group could be due to minification effect of spectacles correction for larger myopic refractive error. LASIK can correct cylindrical component of refraction up to 6 D and non-toric Artisan phakic lenses have only spherical powers. Relaxing incision with concomitant counterquadrant compression sutures were performed to reduce preoperative astigmatism to ≤6 D in the LASIK group and to ≤4 D in the Artisan group, which explains the significant difference in baseline astigmatism between two groups.

The results of our study demonstrate no significant change in UDVA after LASIK. None of the eyes in this group had UDVA ≥20/40 postoperatively. However, UDVA significantly improved after Artisan implantation and 62.5% of the eyes had UDVA of ≥20/40 at the final visit. In addition, the Artisan group had a lower postoperative refractive error compared to LASIK despite higher preoperative refractive error; 6.3% versus 58.8% of eyes were within ±2 D of emmetropia after LASIK and Artisan implantation, respectively. Postoperative CDVA significantly decreased in the LASIK group, whereas it remained stable in the Artisan group at the final follow-up examination compared to preoperative values. Artisan group had a greater gain of CDVA, probably due to retinal image magnification after correcting a high level of myopia. Loss of CDVA in the LASIK group could be due to irregular astigmatism caused by surgery.

Literature review suggests less favorable outcomes with LASIK in our study. Reported UDVA ≥20/40 and CDVA ≥20/40 after LASIK in post-PK eyes varies from 28 to 86% and 74 to 100%, respectively [6–17], in contrast to 0 and 87.5% in our study. Similarly, the loss of 2 lines of CDVA in 18.8% of the LASIK-treated eyes in our series is higher than that reported by other studies (0 to 16%) [7–17]. An improvement of 2-line or greater of CDVA was not observed in our LASIK eyes, in contrast to the reported range of 5 to 56% [6, 8]. The reported reduction in spherical equivalent refraction ranges from 74.8 to 92.9% and in cylinder is 42 to 87.9% in the postkeratoplasty LASIK series [9, 10, 19]. We observed a 40% reduction in spherical equivalent refraction and no change in astigmatism after LASIK. However, most of the LASIK series for postkeratoplasty refractive error had either a short follow-up duration or included PK for variety of etiologies. Our short-term outcomes are in line with the reported efficacy and safety of the LASIK procedure in patients with PK [6–17]. Optimal refractive correction was achieved as early as month-3 post-LASIK in our study, however, our results demonstrated a regression of its effect 3 years postoperatively. There are 3 studies on the long-term outcomes of LASIK for post-PK refractive errors in keratoconus [8, 15, 20]. Kwitko et al. [8] included 14 patients of which 13 had keratoconus, and followed the patients from 12 to 42 months after LASIK. The authors reported 2 lines or more of improvement in the UDVA and CDVA in 64.3 and 21.4% of patients, respectively [8]. Mean reduction in refractive astigmatism was 47.5% with no astigmatism reduction in 28.6% of cases, and progressive changes in refraction were seen in 35.7% postoperatively [8]. In addition, retreatment was necessary in 42.9% of cases in their series [8]. In another study on the 5-year outcomes of LASIK in 30 eyes that had undergone PK for keratoconus, spherical equivalent refraction decreased from − 7.15 D preoperatively to − 0.97 D at postoperative year-1 and -1.05 D at postoperative year-5 [15]. At the last visit, 53.3 and 86.7% of eyes had refractive astigmatism ≤ − 1 D and ≤ − 4 D, respectively [15]. In a similar study, Spadea et al. [20] performed LASIK using standard technique (15 eyes) versus topographically guided two-stage technique (15 eyes) and followed the patients for 36 months. Visual acuity and refraction were significantly improved in both groups, with better outcomes achieved with the two-stage approach [20]. Their visual and refractive outcomes were stable through the course of the study [20]. Progressive changes observed in the long-term follow-up in our study could be due to including recipient corneas within a lamellar flap that was larger than grafts. Lamellar cut of the recipient corneal tissue with the typical altered biomechanics in keratoconus could result in progressive myopia and astigmatism from peripheral corneal ectasia. Another explanation for significant change in post-LASIK refraction could be the small optical zone (5 mm) which could result in regression of astigmatism correction postoperatively.

Our favorable outcomes with phakic Artisan IOL are in line with previous reports. Tahzib et al. [22] implanted Artisan toric phakic IOL in 36 PK eyes for various corneal pathologies including keratoconus (13.6%). After a mean follow up of 28.5 months, spherical equivalent refraction was decreased from − 3.19 ± 4.31 D to − 1.03 ± 1.20 D (77.8% reduction) and refractive astigmatism from − 7.06 ± 2.01 D to − 2.00 ± 1.53 D (88.8% reduction). Postoperative UDVA and CDVA ≥20/40 were achieved in 31.6 and 80.6% of their patients, respectively, and they observed a loss of CDVA of > 2 lines in 8.3% of eyes, and a gain of ≥2 lines in 8.3% [22]. Alfonso et al. [23] implanted posterior chamber spherical (for astigmatism < 2.5 D) or toric (for astigmatism > 2.5 D) implantable Collamer lens (ICL) in 15 PK eyes. At the postoperative month 24, spherical equivalent refraction was decreased from − 9.80 ± 5.88 D to − 0.95 ± 1.12 D. Postoperative UDVA and CDVA ≥20/40 were achieved in 46.6 and 80% of their patients, respectively. No eye lost 1 or more lines and 46% gained 1 or more lines of CDVA, whereas 52% had no change in their CDVA [23]. Iovieno et al. [24] reported the outcome of toric ICL implantation in 7 keratoconus eyes with previous history of PK (*n* = 6) or deep anterior lamellar keratoplasty (*n* = 1). After a mean follow up of 12 months, toric ICL was reported to be very predictable in correcting the spherical refractive error, with a mean postoperative spherical refraction of 0.53 ± 0.75 D. In addition, good result was achieved in astigmatism correction as 71.4% of the eyes had postoperative refractive astigmatism ≤1.5 D. The percentage of eyes with UDVA and CDVA ≥20/40 was 87.5 and 100% in their study [24]. Tahzib et al. [22] and Alfonso et al. [23] observed no regression in spherical equivalent refraction and astigmatism after phakic IOL implantation. Similarly, the stability of the postoperative spherical equivalent and refractive astigmatism was excellent after Artisan lens implantation in our study, yet vector analysis revealed a shift in the axis of the postoperative astigmatism compared to the preoperative value. This shift was probably due to surgically induced astigmatism by placing a 6-mm incision to insert the rigid phakic lens.

The possibility of accelerated endothelial cell attrition of corneal graft is a concern with phakic IOL implantation after keratoplasty. In their series, Tahzib et al. [22] observed 34.8% reduction in endothelial cell count 4 years after Artisan IOL implantation and 3 eyes developed graft failure due to irreversible endothelial rejection (*n* = 2) and progressive endothelial cell loss (*n* = 1) [22]. They included eyes with preoperative endothelial cell count as low as 500 cells/mm^2^. However, we did not observe any graft failure or significant loss of endothelial cells as an endothelial cell count of > 1500 cells/mm^2^ was required prior to phakic IOL implantation. Similarly, Alfonso et al. [23] implanted ICL in eyes with an average preoperative graft endothelial cell density of 1660 ± 427 cell/mm^2^ which was reduced to 1526 ± 398 cell/mm^2^ at 24 months postoperatively (a reduction of 8.1%) [23]. All grafts remained clear in their study at the final visit [23].

There are several limitations to our study. First, we used a spherical phakic IOL because we had no access to the toric model during the study period. Relatively better correction of the astigmatism in the previous similar studies is due to implanting toric IOL [22–24]. Second, we did not evaluate the effect of LASIK procedure on the corneal graft endothelium, and postoperative endothelial cell count was measured 1 year after Artisan implantation; therefore, long-term safety of these procedures with respect to corneal graft endothelium cannot be evaluated by our results. Third, topography was not performed postoperatively to evaluate decentered ablation, irregular astigmatism, or even the recurrence of keratoconus in the LASIK group. However, decentered ablation could not be responsible for increase in postoperative astigmatism in our LASIK group as all patients attained good refractive outcomes up to postoperative month 36. The small sample size could be another limitation of the current study. The result of post hoc power analysis, however, revealed that our study has a power of 95.9% to detect the observed difference in final spherical equivalent refraction between two groups.

## Conclusions

Based on the results of our study, Artisan IOL implantation yields better and stable long-term outcomes compared to LASIK for the management of refractive error after PK for keratoconus. Therefore, phakic IOL implantation has become our primary technique for correction of ametropia for this category of patients when contact lenses or glasses correction is not tolerated. The patients should be counseled that the main objective of this procedure is the reduction of refractive error to a level that allows the use of spectacle correction for the residual ametropia. Phakic toric IOL, which has the substantial advantage of reducing astigmatism, can provide better refractive outcomes.

## Data Availability

The datasets generated and analyzed during the current study are available from the corresponding author on reasonable request.
